# Comparison of intravitreal preservative-free triamcinolone versus posterior sub-tenon triamcinolone acetonide injection for bevacizumab-resistant diabetic macular edema

**DOI:** 10.1186/s12886-024-03291-2

**Published:** 2024-01-19

**Authors:** Seung Hee Jeon, Minhee Kim, Young-Jung Roh

**Affiliations:** 1 Incheon St. Mary’s Hospital, College of Medicine, The Catholic University of Korea, Seoul, Republic of Korea; 2 Department of Ophthalmology and Visual Science, Yeouido St. Mary’s Hospital, College of Medicine, The Catholic University of Korea, 10,63-ro, Yeongdeungpo-gu, Seoul, 07345 Republic of Korea

**Keywords:** Bevacizumab-resistant, Diabetic macular edema, Sub-tenon’s capsule injection, Triamcinolone acetonide

## Abstract

**Background:**

Triamcinolone acetonide (TA) is administered as an intravitreal or posterior sub-Tenon’s capsule injection, as treatment for diabetic macular edema (DME). The intravitreal use of TA is limited because commercially available triamcinolone acetonide contains benzyl alcohol, a neurotoxic preservative. Few studies have compared effects of preservative-free intravitreal TA (IVTA) and posterior sub-Tenon capsule TA (STTA) injections for DME. Thus, herein, we compared the effectiveness of preservative-free IVTA and STTA for treatment of bevacizumab-resistant DME.

**Methods:**

In this retrospective cohort study, bevacizumab-resistant DME was defined as a lack of response to at least three consecutive intravitreal bevacizumab (IVB) injections. Changes in mean central macula thickness (CMT), best-corrected visual acuity (BCVA), and intraocular pressure (IOP) between IVTA and STTA groups were compared at baseline and at 1, 2, and 3 months after treatment.

**Results:**

Forty eyes from 40 patients were included in this study. In the IVTA group, the mean CMT improved significantly from 400.2 ± 144.42 μm at baseline to 288.35 ± 151.74 μm at 3 months after treatment (*p* = 0.01). Similarly, in the STTA group, the mean CMT improved significantly from 446.65 ± 120.74 μm at baseline to 382.9 ± 113.58 μm at 3 months after treatment (*p* = 0.009). The mean BCVA of the IVTA group also showed improvement, decreasing from 0.75 ± 0.55 logarithm of the minimum angle of resolution (logMAR) at baseline to 0.625 ± 0.50 logMAR at 3 months after treatment (*p* = 0.089). Similarly, the mean BCVA of the STTA group improved, from 0.6 ± 0.36 logMAR at baseline to 0.54 ± 0.35 logMAR at 3 months after treatment (*p* = 0.094).

**Conclusion:**

Given that IVTA and STTA demonstrated statistically equivalent anatomical and functional effects in patients with bevacizumab-resistant DME, the less invasive STTA may be considered the preferred treatment approach for the management of bevacizumab-resistant DME.

**Trial registration:**

Retrospectively registered.

## Background

Diabetic macular edema (DME) is a well-known cause of long-term visual impairment in patients with diabetes mellitus (DM) [1]. According to the Early Treatment Diabetic Retinopathy Study (ETDRS), DME is defined as retinal thickening at or within 2 disc-diameters of the macular center, with or without accompanying definitive hard exudates in this area [2].

The pathophysiology of DME is multifactorial and involves a complex interplay between various biochemical, cellular, and molecular processes. Persistent hyperglycemia leads to the loss of pericytes, leukostasis, overexpression of vascular endothelial growth factor (VEGF) and angiotensin II, and the accumulation of advanced glycation end-products, all of which induce vascular inflammation. These changes eventually result in breakdown of the blood–retinal barrier and development of DME [3]. With the increasing life expectancy of the population, the number of patients with diabetes is rising, escalating the importance of DME treatment.

DME treatment has evolved rapidly from the era of laser therapy to the era of anti-VEGF pharmacotherapy. Numerous studies have demonstrated the anatomical and functional efficacy of anti-VEGF agents for the treatment of DME [2, 4–7]. Although monthly injections of anti-VEGF agents have been shown to be effective in previous studies, other strategies, such as pro re nata or treat and extend treatment have been proposed [8].

As steroid therapy can reduce inflammation in DME [9], intravitreal steroids have been used to treat DME [10]. Therefore, when DME persists despite repeated anti-VEGF injections, steroids can be considered an alternative treatment. However, steroids are associated with disadvantages, such as cataracts, increased intraocular pressure (IOP), and glaucoma [11, 12]. Steroids can be administered via three major routes: intravitreal implant, intravitreal injection, and posterior sub-Tenon’s capsule injection. The BEVORDEX trial demonstrated that dexamethasone implants yielded clinical results similar to those of bevacizumab for DME treatment [13]. Triamcinolone acetonide (TA) is used intravitreally [13–15] or via the posterior sub-Tenon capsule [16–18].

Although various long-acting steroid implants have been approved for the treatment of DME, TA is still used intravitreally or as a posterior sub-Tenon’s capsule injection because of its low cost and convenience of injection. The intravitreal use of TA is limited because commercially available triamcinolone acetonide contains benzyl alcohol, a neurotoxic preservative [19]. Few studies have compared the effects of preservative-free intravitreal TA (IVTA) and posterior sub-Tenon’s capsule TA (STTA) injections for DME [20, 21].

In this study, we aimed to compare the effectiveness of preservative-free IVTA and STTA in the treatment of bevacizumab-resistant DME.

## Methods

We reviewed 108 patients who previously underwent IVTA or STTA for bevacizumab-resistant DME between April 2020 and May 2022. In this study, “refractory” DME was defined by the absence of response to three consecutive bevacizumab injections in patients with DME. This retrospective study adhered to the tenets of the Declaration of Helsinki, and the collection of data was carried out in accordance with the approval of the Institutional Review Board of Incheon St. Mary’s Hospital, Korea. All patients provided written informed consent after receiving an explanation of the potential risks associated with IVTA or STTA.

The criteria for participant inclusion in the study were as follows: [1] presence of DME involving the fovea that did not respond to at least three consecutive IVB injections over a period of more than 3–6 months (we defined bevacizumab-resistant DME as a lack of response to IVB if the central macula thickness (CMT) did not decrease after IVB); [2] CMT > 300 μm; [3] absence of steroid use (eye drops or injections); [4] availability of optical coherence tomography (OCT) images for a period of ≥ 3 months after treatment; [5] glycemic levels, indicated by a hemoglobin A1c (HbA1c) of < 8.0%; and [6] no conventional laser treatment in the last 1 year. The criteria for participant exclusion in the study were as follows: [1] presence of other retinal diseases, including age-related macular degeneration, retinal vascular occlusion, polypoidal choroidal vasculopathy, glaucoma, and pathologic myopia; [2] history of vitrectomy; [3] previous ocular surgery, including cataract surgery, within the last 6 months; [4] history of focal laser or panretinal photocoagulation conducted for < 1 year; and [5] previous IVTA or STTA treatment.

To administer IVTA, the patient was placed in a supine position, and topical 0.5% proparacaine (Alcaine, Alcon, Geneve, Switzerland) anesthetic eye drops were applied. We mixed 1 mL of normal saline with preservative-free TA (Maqaid, Wakamoto Pharmaceutical Co., Ltd., Tokyo, Japan) (40 mg/1 bottle), extracted a volume of 0.1 ml (4 mg/0.1 mL) using a 30-gauge needle on a 1-mL syringe, and injected it through the superotemporal pars plana (3.0-mm posterior to the limbus) area. After injection, the patients were prescribed topical 0.5% moxifloxacin eye drops (Vigamox, Novartis, Basel, Switzerland) for 5 days.

To administer STTA, the patient was placed in the supine position, and topical 0.5% proparacaine anesthesia eye drops were applied. A volume of 1.0 mL (40 mg/mL) of TA (DongKwang, Seoul, Korea) was injected into the inferotemporal sub-Tenon’s capsule area using a 30-gauge needle on a 1-mL syringe. After injection, the patients were prescribed topical 0.5% moxifloxacin eye drops for 5 days.

To assess the efficacy of IVTA and STTA, all patients underwent ophthalmological examination, including slit-lamp evaluation, best-corrected visual acuity (BCVA), IOP, and swept-source OCT (Topcon DRI OCT, Topcon, Tokyo, Japan) at baseline and at 1, 2, and 3 months after treatment. BCVA was measured using a standard Snellen chart and was converted to the logarithm of the minimum angle of resolution (logMAR). OCT was performed after pupil dilatation. OCT was used to detect CMT using a macular cube scan protocol (central 6 × 6 mm^2^ area).

### Statistical analysis

Changes in BCVA, CMT, and IOP from baseline to the 1-, 2- and 3-month visits were analyzed using Wilcoxon’s signed-rank test. Changes in CMT and BCVA between the IVTA and STTA groups were assessed using the Mann–Whitney U test. Statistical analyses were conducted using SPSS (version 24.0; SPSS Inc., Chicago, IL, USA).

## Results

Of the 108 participants, 68 were excluded owing to the following reasons: an HbA1c level of > 8% (58 participants), a follow-up period of < 3 months (5 participants), and the presence of an accompanying retinal vein occlusion (5 participants). Finally, 40 individuals were included in this study. Forty eyes of 40 patients who received IVTA (20 eyes of 20 patients, 9 males and 11 females) or STTA (20 eyes of 20 patients, 8 males and 12 females) for the treatment of bevacizumab-resistant DME. The inclusion and exclusion criteria were predefined for participant selection. Patient demographic and disease characteristics are presented in Table 1. No significant differences in demographic and disease characteristics were observed between the IVTA and STTA groups (Table 1). There was no significant difference in the mean CMT at baseline between the STTA and IVTA groups.

**Table 1:** Baseline demographics and characteristics of 40 eyes in 40 patients with bevacizumab-resistant refractory DME

Patient characteristics	IVTA	STTA	P value
**Number of eyes**	20	20	
**Mean age (years)**	66.53 ± 8.19	64 ± 8.63	0.158
**Sex (n, %)**			0.102
Male	9 (45%)	8 (40%)	
Female	11 (55%)	12 (60%)	
**DM duration (years)**	16.95 ± 7.69	15.5 ± 9.29	0.629
**HbA1c (%)**	7.41 ± 1.67	7.56 ± 1.68	0.763
**Number of previous intravitreal bevacizumab injection (n)**	4.05 ± 2.01	4.25 ± 2.9	0.529
**Type of DME (n, %)**			0.151
Diffuse type	18 (90%)	17 (85%)	
Focal type	2 (10%)	3 (15%)	
**Status of diabetic retinopathy (n, %)**			0.568
Moderate NPDR	2 (10%)	3 (15%)	
Severe NPDR	4 (20%)	1 (5%)	
PDR	14 (70%)	16 (80%)	
**Previous conventional laser (n, %)**			0.151
PRP	18 (90%)	17 (85%)	
None	2 (10%)	3 (15%)	
**Status of lens (n, %)**			0.100
Phakic	9 (45%)	10 (50%)	
Pseudophakic	11 (55%)	10 (50%)	
**Mean baseline BCVA (logMAR)**	0.75 ± 0.55	0.6 ± 0.36	0.522
**Mean baseline CMT (µm)**	400.2 ± 144.42	446.65 ± 120.74	0.402
**Mean baseline IOP (mmHg)**	17.2 ± 2.75	17.65 ± 2.37	0.662

BCVA, best-corrected visual acuity; CMT, central macular thickness; DM, diabetes mellitus; DME, diabetic macular edema; IOP, intraocular pressure; IVTA, intravitreal triamcinolone acetonide injection; NPDR, non-proliferative diabetic retinopathy; PDR, proliferative diabetic retinopathy; PRP, panretinal photocoagulation; SRD, serous retinal detachment; STTA, sub-Tenon’s capsule triamcinolone acetonide injection

* *p* < 0.05

**Fig. 1:**
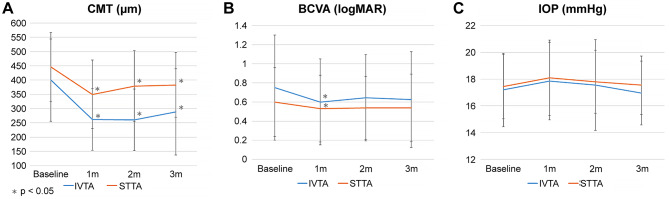
Mean CMT, BCVA, and IOP with intravitreal and sub-Tenon’s capsule triamcinolone acetonide injection. Values were measured at baseline and at 1, 2 and 3 months after treatment. BCVA, best-corrected visual acuity; CMT, central macular thickness; IOP, intraocular pressure

**Fig. 2:**
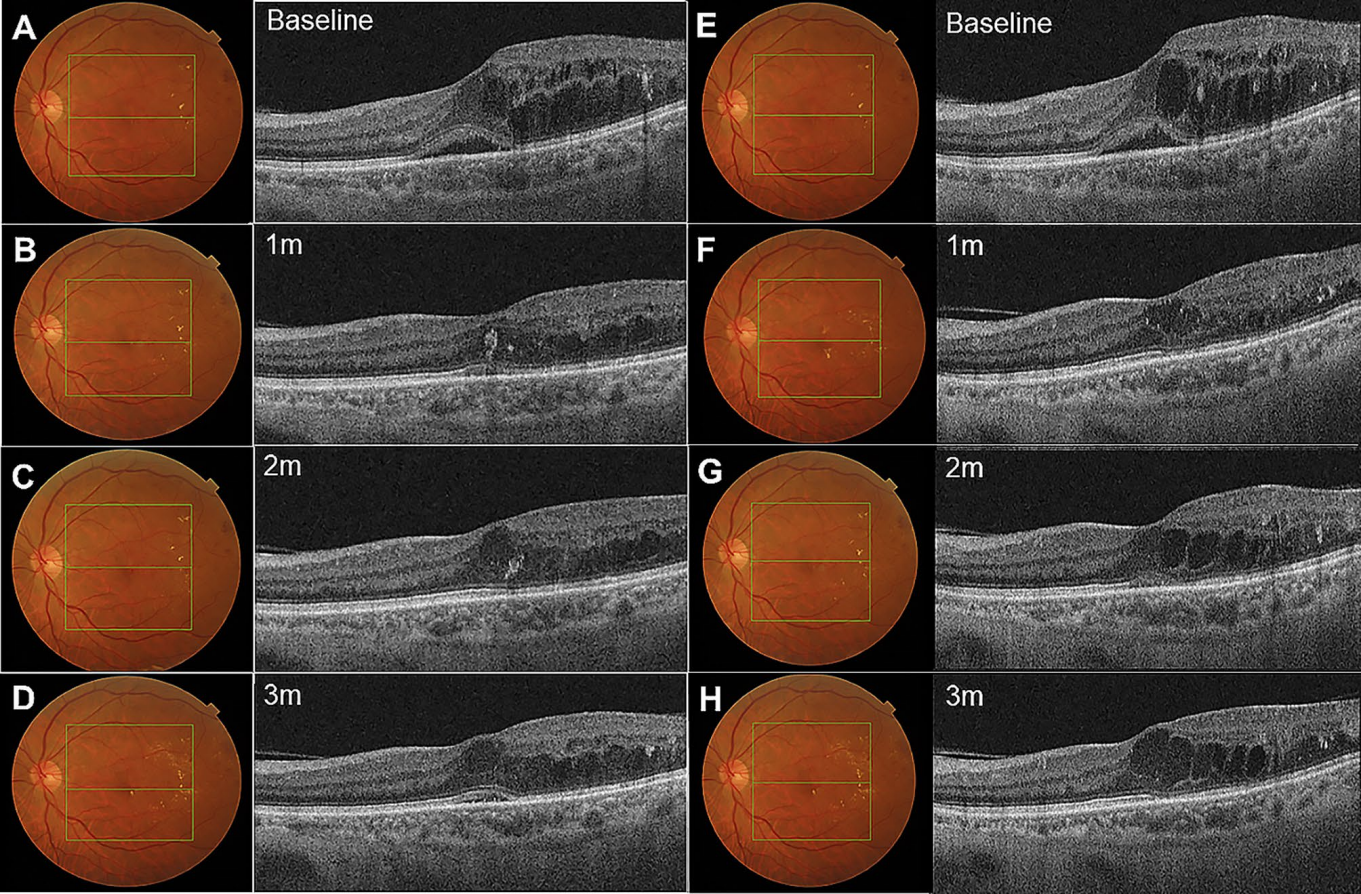
Representative images comparing results of IVTA and posterior STTA injections in patients with bevacizumab-resistant DME. (**A**) Initial presentation of the left eye of a 59-year-old male patient who showed no response to IVB injections. SD-OCT reveals diffuse retinal thickening and serous retinal detachment associated with diabetic macular edema. (**B**) Resolution of macular edema and serous retinal detachment at 1 month after IVTA injection. (**C**) Complete resolution of serous retinal detachment and cystic change at 2 months after treatment. (**D**) Slight aggravation of serous retinal detachment and macular edema at 3 months after treatment. (**E**) Initial presentation of the left eye of a 57-year-old male patient who showed no response to IVB injections. SD-OCT shows diffuse retinal thickening and serous retinal detachment associated with diabetic macular edema. (**F**) Complete resolution of serous retinal detachment and macular edema at 1 month after posterior STTA injection. (**G**) Sustained macular edema at 2 months after treatment. (**H**) Slight aggravation of macular edema at 3 months after treatment. DME, diabetic macular edema; IVB, intravitreal bevacizumab; IVTA, intravitreal triamcinolone acetonide; SD-OCT, Spectral-domain optical coherence tomography; STTA, sub-Tenon’s capsule triamcinolone acetonide

The mean CMT of both the IVTA group and the STTA group improved significantly over the course of 3 months (Table 2). The mean BCVA of the IVTA group improved from 0.75 ± 0.55 logMAR at baseline to 0.6 ± 0.45 logMAR at 1 month after treatment (*p* = 0.036), while the mean BCVA of the STTA group improved from 0.6 ± 0.36 logMAR at baseline to 0.53 ± 0.35 logMAR at 1 month after treatment (*p* = 0.016) (Fig. 1). Even though seven of the 40 eyes required anti-glaucoma drugs, no significant changes in the mean IOP of both groups were noted at 1, 2, and 3 months after treatment as compared to baseline (Table 2). SD-OCT images of representative patients are presented in Fig. 2.

**Table 2:** Changes in central macular thickness, best-corrected visual acuity, and intraocular pressure observed during the 3-month follow-up period in each group: intravitreal and sub-Tenon’s capsule triamcinolone acetonide injection

	IVTA	P value	STTA	P value	Differences between IVTA and STTA P value
**CMT (µm)**					
Baseline	400.2 ± 144.42		446.65 ± 120.74		
1 month	261.4 ± 108.18	< 0.001*	349.9 ± 120.15	0.005*	0.194
2 months	260.45 ± 108.20	< 0.001*	379 ± 124.11	0.035*	0.168
3 months	288.35 ± 151.74	0.01*	382.9 ± 113.58	0.009*	0.903
**BCVA (logMAR)**					
Baseline	0.75 ± 0.55		0.6 ± 0.36		
1 month	0.6 ± 0.45	0.036*	0.53 ± 0.35	0.016*	0.546
2 months	0.645 ± 0.45	0.199	0.54 ± 0.33	0.076	0.900
3 months	0.625 ± 0.50	0.089	0.54 ± 0.35	0.094	0.787
**IOP (mmHg)**					
Baseline	17.2 ± 2.75		17.45 ± 2.41		
1 month	17.85 ± 2.9	0.129	18.10 ± 2.82	0.106	0.754
2 months	17.55 ± 3.38	0.721	17.80 ± 2.35	0.384	0.575
3 months	16.95 ± 2.39	0.981	17.55 ± 2.18	0.872	0.342

BCVA, best-corrected visual acuity; CMT, central macular thickness; IOP, intraocular pressure; IVTA, intravitreal triamcinolone acetonide injection; STTA, Sub-tenon capsule triamcinolone acetonide injection

* *p* < 0.05

Comparing the changes in CMT and BCVA between the IVTA and STTA groups at baseline and at 1, 2, and 3 months of treatment, no statistically significant difference was observed (Table 2).

Additionally, 1 month after injection, four patients in the IVTA group and three patients in the STTA group experienced a minor elevation in IOP, ranging from 22 to 25 mmHg. Nevertheless, all patients showed a return to normal IOP after the administration of a combination of topical carbonic anhydrase inhibitors and beta-blocker eye drops. No injection-related adverse events, such as secondary glaucoma, sterile or infectious endophthalmitis, and retinal detachment, were observed during the 3-month follow-up period.

## Discussion

In this study, we demonstrated that preservative-free IVTA and STTA exhibited equivalent anatomical and functional effects in bevacizumab-resistant DME. Although no statistically significant differences were observed between the two groups, when observed over the course of 3 months after a single injection, IVTA exhibited approximately a 60-µm greater reduction in CMT and showed an improvement in BCVA of approximately 0.06 logMAR, equivalent to around three additional ETDRS letters. Increased IOP was noted in four cases in the IVTA group and in three cases in the STTA group. Therefore, the proportions of patients using anti-glaucoma medication were similar between the two groups. While the mean IOP in the IVTA group decreased slightly, the mean IOP increased in the STTA group. In the IVTA group, the mean IOP decreased by 0.25 mmHg at 3 months compared to baseline, which may have been due to the use of anti-glaucoma medication. In the STTA group, on the other hand, the mean IOP increased by 0.1 mmHg at 3 months compared to baseline. Although the change in mean IOP was not statistically significant (*p* = 0.342), the increase in IOP was numerically greater in the STTA group than in the IVTA group. Therefore, in patients with bevacizumab-resistant DME, both IVTA and STTA were effective. Although differences were not statistically significant, IVTA showed better reduction in CMT, better BCVA improvement, and a decrease in IOP compared to STTA.

Steroids have been used to treat DME owing to their anti-inflammatory mechanisms, inhibition of VEGF synthesis, and stabilization of vascular hyper-permeability [22–25]. Among the various steroids, TA is widely used for DME treatment because of its excellent anti-angiogenic and anti-inflammatory properties and its ability to stabilize blood–retinal barrier effects [26]. In real-world settings of DME treatment, switching to steroids for cases resistant to anti-VEGF therapy is a commonly used and effective strategy, with favorable results [27, 28]. In addition, STTA has also been reported to be effective in treating other diseases causing cystoid macular oedema, such as Irvine-Gass syndrome [29].

Both IVTA and STTA commonly use high local steroid concentrations, minimizing the risk of systemic side effects. Various studies have compared the efficacy of IVTA and STTA in patients with DME. Previous systematic reviews have indicated that, in a short-term follow-up period of 3 months, IVTA generally outperformed STTA in terms of CMT and BCVA [26]. However, no previous study has compared bevacizumab-resistant DME focusing on a limited number of patients with long-term unresponsiveness to anti-VEGF therapy. Interestingly, our findings revealed no significant differences in the effects of IVTA and STTA. This may be attributable to the underlying pathogenesis of DME. The lack of response to anti-VEGF therapy suggested the presence of low VEGF levels.

Similar to previous studies, our study demonstrated that both IVTA and STTA were most effective in terms of CMT reduction during the first month after injection, with effectiveness gradually diminishing thereafter [20]. However, in terms of BCVA, a beneficial effect was observed only in the first month after treatment with no subsequent improvement, indicating that the functional effect was not as pronounced as the anatomical effect.

Inoue et al. recently reported that IVTA injection results in significantly higher vitreous concentrations of the steroid (1.22 ± 0.24 µg/ml) compared to STTA injection (< 0.001 µg/ml) [30]. IVTA directly delivers the drug, whereas STTA passes through the choroid and sclera differently. This difference in the administration routes can lead to inadequate drug delivery. Owing to the higher concentration of IVTA, the incidence of side effects such as intraocular pressure elevation, glaucoma, and cataracts has been found to be higher with IVTA than with STTA [31, 32]. However, in this study, STTA showed a similar effect to IVTA, despite its lower concentration, which suggests that the integrity of the blood–retinal barrier may have been compromised, leading to a reduced therapeutic effect.

Pharmacologic studies have demonstrated that after IVTA injection, the drug concentration is high at 1 month, resulting in effective outcomes, and that this effect is maintained for up to 3 months [33]. Similarly, animal model experiments have demonstrated that the effect of STTA was maintained pharmacokinetically for months [34]. Therefore, side effects, such as elevated IOP, can be attributed most to the first month. In our study, 7 of 40 patients (17.5%) showed an increased IOP in the first month after injection. This is similar to the previously reported occurrence rate of elevated IOP after steroid injection, which ranged from 10 to 30% [35–37].

There are several limitations in our study. First, we employed a retrospective, nonrandomized study design. Second, the inclusion of only a small number of cases may have affected the generalizability of our findings. Third, although all patients received at least three consecutive bevacizumab injections before steroid injection, other anti-VEGF agents may have been considered for the treatment of bevacizumab-resistant DME. Lastly, due to the short observation period, we were unable to ascertain long-term complications, such as the potential occurrence of severely increased IOP > 25 mmHg, secondary glaucoma, or cataract, which are common side effects of steroid injections.

## Conclusions

In this study, we demonstrated that IVTA and STTA exhibited statistically equivalent anatomical and functional effects in patients with bevacizumab-resistant DME. Given that STTA is less invasive compared with IVTA, STTA may be considered a preferred treatment approach for the management of DME. However, conducting a future study with a larger sample size and long-term follow-up would yield valuable insights into this topic.

## Data Availability

The datasets used and/or analysed during the current study available from the corresponding author on reasonable request.

## Abbreviations

BCVA: best-corrected visual acuity
CMT: central macula thickness
DM: diabetes mellitus
DME: diabetic macular edema
ETDRS: Early Treatment Diabetic Retinopathy Study
HbA1c: hemoglobin A1c
IOP: intraocular pressure
IVB: intravitreal bevacizumab
IVTA: preservative-free intravitreal TA
logMAR: logarithm of the minimum angle of resolution
OCT: optical coherence tomography
STTA: posterior sub-Tenon capsule TA
TA: triamcinolone acetonide
VEGF: vascular endothelial growth factor
